# Molecular Characterization of Divergent Closterovirus Isolates Infecting *Ribes* Species

**DOI:** 10.3390/v10070369

**Published:** 2018-07-12

**Authors:** Igor Koloniuk, Thanuja Thekke-Veetil, Jean-Sébastien Reynard, Irena Mavrič Pleško, Jaroslava Přibylová, Justine Brodard, Isabelle Kellenberger, Tatiana Sarkisova, Josef Špak, Janja Lamovšek, Sebastien Massart, Thien Ho, Joseph D. Postman, Ioannis E. Tzanetakis

**Affiliations:** 1 Department of Plant Virology, Institute of Plant Molecular Biology, Biology Centre of the Academy of Sciences of the Czech Republic, v.v.i., Branišovská 31, 370 05 České Budějovice, Czech Republic; koloniuk@umbr.cas.cz (I.K.); pribyl@umbr.cas.cz (J.P.); sarkisova@umbr.cas.cz (T.S.); spak@umbr.cas.cz (J.Š.); 2 Department of Plant Pathology, Division of Agriculture, University of Arkansas System, Fayetteville, AR 72701, USA; thanujatv@gmail.com (T.T.-V.); txho@uark.edu (T.H.); itzaneta@uark.edu (I.E.T.); 3 Virology-Phytoplasmology Laboratory, Agroscope, 1260 Nyon, Switzerland; jean-sebastien.reynard@agroscope.admin.ch (J.-S.R.); justine.brodard@agroscope.admin.ch (J.B.); isabelle.kellenberger@agroscope.admin.ch (I.K.); 4 Agricultural Institute of Slovenia, Hacquetova ulica 17, 1000 Ljubljana, Slovenia; irena.mavricplesko@kis.si (I.M.P.); janja.lamovsek@kis.si (J.L.); 5 Plant Pathology Laboratory, TERRA-Gembloux Agro-Bio Tech, University of Liège, Passage des Déportés, 2, 5030 Gembloux, Belgium; sebastien.massart@uliege.be; 6 National Clonal Germplasm Repository, United States Department of Agriculture, Corvallis, OR 97333, USA; joseph.postman@ars.usda.gov

**Keywords:** *Ribes*, currant, closterovirus, recombinants/recombination

## Abstract

Five isolates of a new member of the family *Closteroviridae*, tentatively named blackcurrant leafroll-associated virus 1 (BcLRaV-1), were identified in the currant. The 17-kb-long genome codes for 10 putative proteins. The replication-associated polyprotein has several functional domains, including papain-like proteases, methyltransferase, Zemlya, helicase, and RNA-dependent RNA polymerase. Additional open reading frames code for a small protein predicted to integrate into the host cell wall, a heat-shock protein 70 homolog, a heat-shock protein 90 homolog, two coat proteins, and three proteins of unknown functions. Phylogenetic analysis showed that BcLRaV-1 is related to members of the genus *Closterovirus*, whereas recombination analysis provided evidence of intraspecies recombination.

## 1. Introduction

Black and red currants (*Ribes* species (spp.)) are economically important berry crops. They are deciduous, unarmed shrubs native to the northern latitudes of Asia, Europe, and North America, and they belong to the subgenera *Coreosma* and *Ribesia* of the genus *Ribes* [1]. The genus includes more than 150 diploid species and numerous cultivated varieties [2]. Diseases caused by viruses and virus-like agents have been studied in currants from the beginning of last century [3,4,5]. New currant viruses were recently identified using traditional methods or high-throughput sequencing (HTS) [6,7,8,9,10,11].

Notwithstanding the progress in currant virology, there are gaps in the knowledge, one of which is addressed here through the characterization of a new closterovirus complex, affecting both black and red currants. Roberts and Jones observed closterovirus-like particles in *Ribes* in 1997 [12]. In 2010, Besse et al. observed similar particles in currants showing downward leaf rolling and interveinal reddening in the summer and autumn [6]. They produced antisera for serological detection, and designed primers allowing for the detection of two molecular variants of this virus. In 2015, Ho et al. reported a closterovirus in black currant in the USA and developed a molecular diagnostic assay for its detection [13].

The family *Closteroviridae* includes the genera *Ampelovirus*, *Closterovirus*, *Crinivirus*, and *Velarivirus*, with vectors ranging from mealybugs and soft scales to aphids and whiteflies [14,15]. The genome segments are encapsidated by two coat proteins (CPs) in characteristically long, flexuous particles [14]. Closterovirids have a five-gene block involved in virion assembly and movement that, in addition to two CPs, includes a small transmembrane protein, a ~60 kDa protein, and a HSP70 homolog [15]. Their host range is usually narrow, but the acquisition of accessory genes is believed to play a role in host-range expansion [15].

Here, we studied in depth a closterovirus species, tentatively named blackcurrant leafroll associated virus 1 (BcLRaV-1), identified in black and red currants including particle morphology, genome organization, and the evolutionary forces acting on the virus.

## 2. Materials and Methods

### 2.1. Transmission Electron Microscopy

Virus particles from isolate BC28074 were purified as described by Gugerli and Ramel [16] and observed using a Tecnai Spirit transmission electron microscope (TEM).

### 2.2. Genome Assembly and Organization

The genome of all isolates was obtained using a combination of HTS and Sanger sequencing in four labs and sequences deposited in GenBank (Table 1).

GR and G55: Four red currant accessions were extracted with the GeneJET Plant RNA Purification Kit (Thermo Fisher Scientific, Vilnius, Lithuania) and mRNA-enriched (TruSeq Stranded mRNA kit, Illumina, San Diego, CA, USA) before being subjected to HTS (SeqMe s.r.o., Dobříš, Czech Republic). Missing sequence segments were obtained by PCR amplification using the Q5 High-Fidelity Master Mix (NEB, Ipswich, MA, USA). The 5′-termini were completed and sequenced with a 5′ rapid amplification of complementary DNA (cDNA) ends (RACE) kit (Invitrogen, Carlsbad, CA, USA), and the 3′-ends were derived as previously described [17]. Sequence verification and gap-filling were done through Sanger sequencing of PCR amplicons or cloned into a pGEM T-Easy vector system (Promega, Road Madison, WI, USA).

SLO: Total RNA was extracted from 100 mg of leaf tissue using an RNeasy Plant Mini Kit (Qiagen, Sverige, Denmark), in which RLT buffer was supplemented with a 10% Plant RNA Isolation Aid (Thermo Fisher Scientific). The extracted total RNA was quantified on a Bioanalyzer 2000. Ribosomal RNA was depleted using a RiboMinus Plant Kit for RNA-Seq (Thermo Fisher Scientific), and total RNA libraries were then prepared following the manufacturer’s instructions for a TrueSeq Stranded mRNA kit (Illumina), without the poly-A enrichment step. The RNA libraries were sequenced on a Nextseq 500 sequencing machine at the Liege University in Belgium, with a read length of 2 × 150 nt. (Etiology fair COST Divas). Missing sequence fragments were PCR amplified using the Phusion Flash High Fidelity Master Mix (Thermo Fisher Scientific), and the PCR products were directly sequenced (Macrogen, Seoul, Korea).

BC28074: Virus particles were purified from mature leaves as previously described [18]. Subsequently, RNA was extracted using an RNeasy Plant Mini kit (Qiagen, Hilden, Germany). The library was prepared using the TrueSeq Stranded mRNA kit (Illumina) following the manufacturer’s instructions and subjected to HTS on a HiSeq 4000 (Fasteris SA, Geneva, Switzerland). The 5′- and 3′-terminal sequences of BC28074 were obtained using a RACE system for the rapid amplification of cDNA ends (Invitrogen). At least two PCR amplicons were cloned and Sanger-sequenced.

US: HTS was performed on degenerate oligonucleotide-primed reverse-transcription-PCR (DOP RT-PCR) products derived from double-stranded RNA-enriched (dsRNA) material of the infected plant, following the procedures described previously [7]. Missing genome fragments were obtained via RT-PCR using virus-specific primers. The 5′-terminal sequences were obtained using a FirstChoice RLM-RACE Kit (Thermo Fisher Scientific), whereas the 3′-ends were obtained using RACE–RT-PCR on polyadenylated RNAs (Poly (A) Tailing Kit, Applied Biosystems, Foster City, CA, USA). All PCR products were sequenced so as to achieve at least three-fold coverage of the regions.

### 2.3. In Silico Analyses

Sequence analyses were done using a CLC Genomics Workbench 9.5.1 (Qiagen) and the Geneious 9.1.5 software (Biomatters Limited, Auckland, New Zealand). Transmembrane prediction was carried out using the TMHMM 2.0c tool (http://www.cbs.dtu.dk/services/TMHMM/). Multiple sequence alignments were built with the Multiple Alignment using Fast Fourier Transform (MAFFT) program [19]. Phylogeny reconstructions were inferred using the maximum-likelihood method with an approximate likelihood ratio test for branches. The phylogenetic trees were visualized using the Interactive Tree of Life v3 tool [20]. Putative recombination events were detected and evaluated in the RDP4 program [21], using a MAFFT-built multiple alignment of the complete genome sequences.

## 3. Results and Discussion

### 3.1. Sequence and Genome Organization

**Figure 1:**
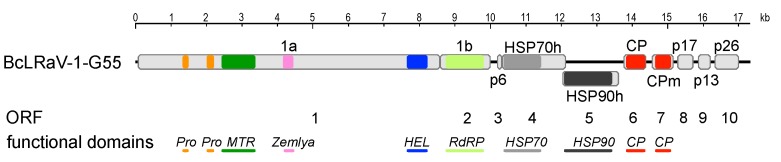
Schematic representation of the genomic organization of the Gabreta 55 (G55) isolate of blackcurrant leafroll associated virus 1 (BcLRaV-1-G55). The genome is drawn as a black line, and the predicted open reading frames (ORFs) are represented by shaded rectangles. Annotations, ORF numbers, and identified functional domains are given below. Abbreviations: Pro—papain-like leader proteinase, Zemlya—see text for description, MTR—methyltransferase, HEL—helicase, RdRP—RNA-dependent RNA polymerase, HSP70—heat-shock protein 70, HSP90—heat-shock protein 90, CP— capsid protein, and CPm—minor capsid protein.

The genomes of five isolates from Europe and North America, infecting both black and red currants, were reconstructed (Table 1). The genome lengths ranged from 16,996 to 17,313 nucleotides (nt) and coded for 10 open reading frames (ORFs; Figure 1 and Table 2), with genome organization being identical among isolates. The results of the 3′-RACE with virus-specific primers on GR and G55 suggested an absence of the poly(A) tail at the 3′-terminus, similar to other closteroviruses, and therefore the analysis was not repeated for the other isolates.

ORFs 1 and 2 encode the replication-associated proteins (Table 2), in which ORF2 is presumably translated via a +1 ribosomal frameshift from ORF1, a mechanism prevalent in closteroviruses [22], resulting in a fusion polyprotein 1a/1b. The sequence surrounding the potential ribosome +1 slippage site is conserved in all isolates: cg(a/g/c)guuUAAcua (the stop codon of ORF1 is capitalized; the first proposed codon of ORF2 is underlined). A conserved domain search identified five replication-associated domains in the 1a/1b protein (Figure 1). Two copies of a papain-like leader proteinase (Pro; pfam05533) were found upstream of a methyltransferase motif (MTR; pfam01660). The copies were diverse, sharing only 21% to 30% amino acid (aa) identity within each isolate. While intragenome duplication of coat proteins is a fairly common feature of family members, two copies of the leader protease are present in some members of the genus *Closterovirus*. Duplication events are independent across species, followed by the functional divergence of each copy [23]. The roles of previously studied viral leader proteases are not only limited to self-processing (proteolysis), but also include the regulation of genome replication and transcription [24]. Host-specific effects were demonstrated for leader proteinases of grapevine leafroll-associated virus-2 (GLRaV-2) and particularly suggested that such diversification is needed for a closterovirus infection of perennial and/or woody plants [24].

**Figure 2:**
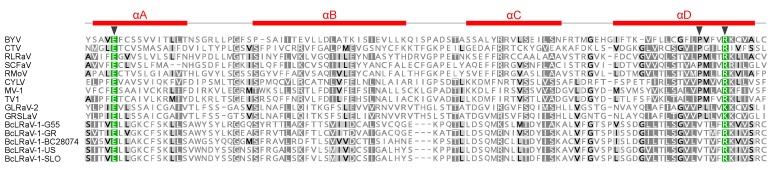
Multiple alignment of the Zemlya-region sequences of beet yellows virus (BYV, NC_001598), citrus tristeza virus (CTV, AB046398), rose leaf rosette-associated virus (RLRaV, NC_024906), strawberry chlorotic fleck-associated virus (SCFaV, NC_008366), raspberry mottle virus (RMoV, NC_008585), carrot yellow leaf virus (CYLV, NC_013007), mint virus 1 (MV-1, NC_006944), tobacco virus 1 (TV1, NC_027712), grapevine leafroll-associated virus 2 (GLRaV-2, NC_007448), grapevine rootstock stem lesion-associated virus (GRSLaV, NC_004724), BcLRaV-1-G55 (MH460557), BcLRaV-1-GR (MH460558), BcLRaV-1-BC28074 (MH541840), BcLRaV-1-US (MH500053), and BcLRaV-1-SLO (MH480582). Gaps are shown as dashes. Black triangles indicate conserved positions. Previously predicted [25] secondary structure is shown above the alignment.

A recently described “Zemlya” region was identified after the MTR domain (Figure 1), and presumably guides the remodeling of the endoplasmic reticulum membranes during infection, a process connected to the formation of viral replication factories [25]. The Zemlya region was predicted to form four α-helices, and three strictly conserved positions were found in known closteroviruses [25]. The BcLRaV-1 isolates differed in one of the conserved positions, featuring a valine instead of a proline residue (Figure 2). This change is noteworthy, as the αD region was predicted to form an amphipathic helix, and the proline, being strongly disfavored in helices, could induce a kink in the helix [25].

The C-proximal part of the 1a/1b protein contains a viral helicase (HEL; superfamily 1, pfam01443) and an RNA-dependent RNA polymerase (RdRP; pfam00978). Together with the MTR domain located at the 1a/1b N-terminus, they constitute a replication module conserved across the entire alphavirus superfamily [26]. In other closteroviruses, a large region between the MTR and HEL domains is believed to be cleaved by either an unidentified viral or cellular protease [27]. The putative ORF3 encodes a p6 protein with a predicted transmembrane domain. The p6 counterpart in beet yellows virus (BYV) is associated with the endoplasmic reticulum, and it functions as a cell-to-cell movement protein [28]. It is separated by a short intergenic region from the putative heat-shock protein 70 homolog (HSP70h; cd10170). The HSP70h of BYV and other closteroviruses is an integral part of the virion, and it plays a role in cell-to-cell movement through its ATPase activity [15]. ORF5, coding for an HSP90h-like protein (pfam03225), partially overlaps the 3′-proximal region of ORF4. Two putative structural proteins, the major and minor capsid proteins (CP and CPm, respectively; (pfam01785)), are encoded by ORF6 and ORF7, respectively. The closterovirus CPm was shown to be essential for encapsidation of the 5′-region of the viral RNA. Downstream of the capsid proteins, closteroviruses encode a variable number of accessory proteins, and their functionality was determined only for some. For example, the p20 and p21 of BYV participate in systemic transport and the suppression of RNA silencing, respectively [22]. In the citrus tristeza virus (CTV), p23, a suppressor of RNA silencing, did not have any identifiable orthologs in other closteroviruses [29]. Similar to the majority of the studied closterovirids, the three predicted ORFs downstream of the capsid proteins (p17, p13, and p26) did not have significant (E-value cut-off: 10^−3^) similarity to other viral proteins and do not contain transmembrane domains.

### 3.2. Divergence of BcLRaV-1

**Figure 3:**
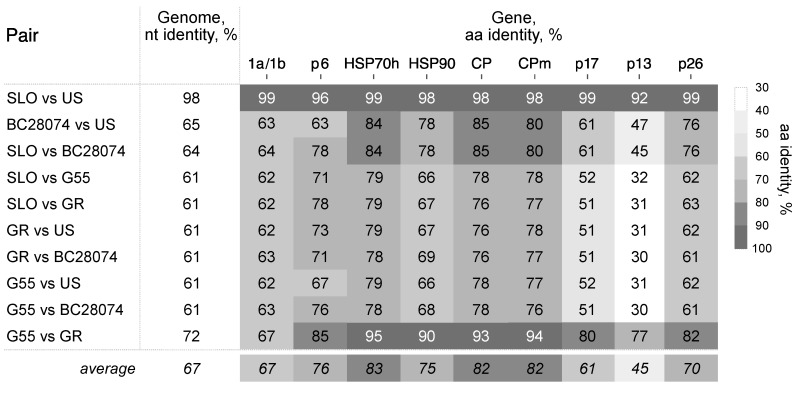
Pairwise nucleotide and predicted amino acid (aa) protein identities among BcLRaV-1 isolates.

Nucleotide divergence between the isolates reached 39% (Figure 3). The black and red currant isolates showed divergence of 35% and 29% among them, respectively. For individual proteins, identities ranged from 45% for p13 to 83% for HSP70h.

Noticeably, neither the predicted 1b, HSP70h, nor the CP proteins showed more than 25% diversity, the species demarcation identity criteria for closteroviruses [15]. No two isolates, except SLO and US, shared no more than 90% amino acid identities across genes. The isolates infecting the red currant were more than 80% identical, with the exception of p13.

**Figure 4:**
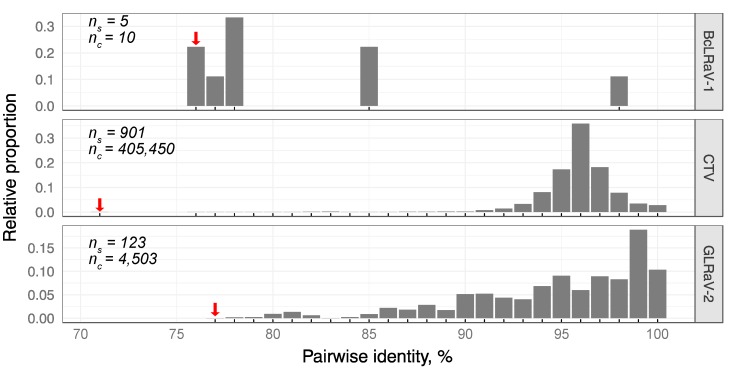
Distribution of the pairwise amino acid CP sequence homologies of BcLRaV-1, GLRaV-2, and CTV isolates. Complete protein sequences were obtained from GenBank (June 2018). The number of analyzed sequences (ns) and their pairwise combinations (nc) are shown. The lowest identity value is indicted by a red arrow. Additionally, the positions of the data points along the *x* axes are denoted by tick marks.

The divergence of BcLRaV-1 isolates resembles those observed in grapevine leafroll-associated viruses 3 and 4, members of the genus *Ampelovirus* [30,31], with values of 62% and 68%, respectively. For members of the genus *Closterovirus*, the most distant examples could be found among CTV and GLRaV-2, with isolates sharing 79% and 72% nt identities, respectively. Analysis of the CP aa homologies among CTV and GLRaV-2 isolates revealed divergence comparable to BcLRaV-1 (Figure 4). A unimodal distribution was observed with peaks of 96% and 99% for CTV and GLRaV-2, respectively, whereas the BCLRaV-1 profile differed, with the majority of values being in the 76–77% region. It should be noted that the BcLRaV-1 analysis is only based on five isolates, unlike the other two viruses, where the analyses were based on hundreds.

Interestingly, the 5′- and 3′-untranslated regions (UTRs) showed considerable divergence, with 65–81% and 56–76% of positions being conserved, respectively. For comparison, CTV isolates show only 60–70% nt identity in 5′-UTRs [32].

### 3.3. Phylogenetic Analysis

**Figure 5:**
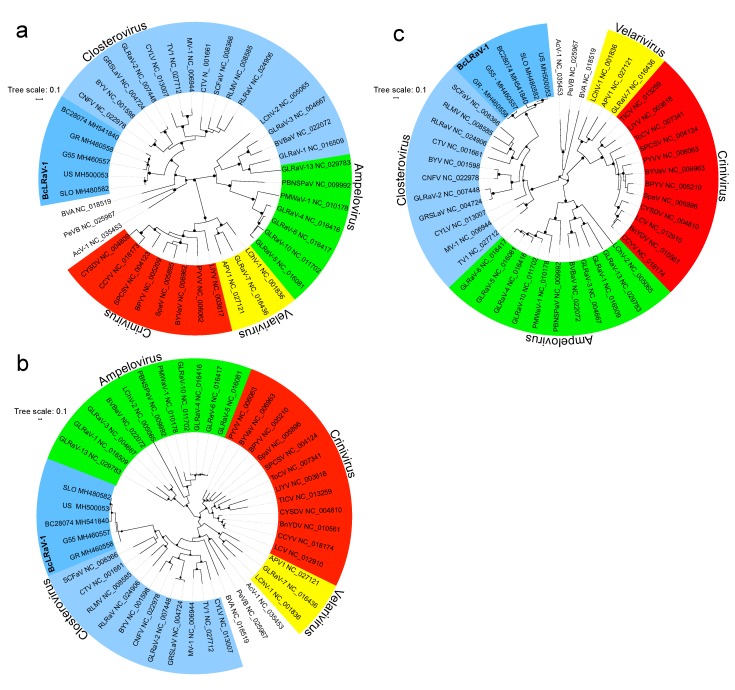
Phylogenetic analysis of the aligned amino acid sequences of (**a**) the polymerase (ORF2, 1b), (**b**) the coat protein (ORF6), and (**c**) HSP70h (ORF4). Branching support with values more than 80% is indicated by dots. Amino acid sequences used in the analysis were obtained from Genbank for the following members of the family *Closteroviridae*: actinidia virus 1 (AcV-1), areca palm velarivirus 1 (APV1), bean yellow disorder virus (BnYDV), beet pseudoyellows virus (BPYV), blackberry vein banding-associated virus (BVBaV), blackberry yellow vein-associated virus (BYVaV), blueberry virus A (BVA), carnation yellow fleck virus (CNFV), cucurbit chlorotic yellows virus (CCYV), cucurbit yellow stunting disorder virus (CYSDV), grapevine leafroll-associated virus 1 (GLRaV-1), grapevine leafroll-associated virus 3 (GLRaV-3), grapevine leafroll-associated virus 4 (GLRaV-4), grapevine leafroll-associated virus 5 (GLRaV-5), grapevine leafroll-associated virus 6 (GLRaV-6), grapevine leafroll-associated virus 7 (GLRaV-7), grapevine leafroll-associated virus 10 (GLRaV-10), grapevine leafroll-associated virus 13 (GLRaV-13), lettuce chlorosis virus (LCV), lettuce infectious yellows virus (LIYV), little cherry virus 1 (LChV-1), little cherry virus 2 (LChV-2), persimmon virus B (PeVB), pineapple mealybug wilt-associated virus 1 (PMWaV-1), plum bark necrosis stem pitting-associated virus (PBNSPaV), potato yellow vein virus (PYVV), raspberry leaf mottle virus (RLMV), strawberry pallidosis-associated virus (SpaV), sweet potato chlorotic stunt virus (SPCSV), tomato chlorosis virus (ToCV), tomato infectious chlorosis virus (TICV).

A maximum likelihood phylogenetic inference of the aa sequences of 1b and CP of the five isolates and representative members of the family confirmed the taxonomical status of BcLRaV-1 in the *Closterovirus* genus (Figure 5). Phylogenetic trees based on the 1b and CP sequences (Figure 5a,b) showed a clear separation of BcLRaV-1 from other members of the genus, whereas analysis based on the HSP70h sequences (Figure 5c) supported its clustering with strawberry chlorotic fleck-associated virus, raspberry leaf mottle virus, rose leaf rosette-associated virus, and CTV. The branching topology of the BcLRaV-1 isolates showed some discrepancy. Black and red currant isolates were clustered separately in the CP and HSP70h trees (Figure 5b,c), but were mixed in the 1b tree, with BC28074 grouping with the red currant isolates (Figure 5a). The fact that divergent topologies produced the different genes pointed to recombination. To test this hypothesis, a recombination analysis was performed.

### 3.4. Recombination Analysis

Recombination events supported by at least six of the nine algorithms applied in RDP4 [21] were considered as possible events (Table 3; complete list in shown in Supplementary Material). Significant evidence of three events was found (Table 3). For the US isolate, the recombinant region covered a part of ORF2 (1b protein) and stretched to ORF3 (p6 protein), involving the BC28074 and GR lineages as major and minor parents, respectively. In contrast, G55 is a product of a recombination event between the US and GR lineages, with a predicted recombinant area covering almost the entirety of the 1a/1b coding area (Table 3).

Recombination is one of the mechanisms facilitating viral evolution. For several closteroviruses and ampeloviruses, recombination events have been identified [14,33]. Isolates from black or red currant were involved in the recombination process, suggesting a complex evolutionary history for BcLRaV-1. The number of analyzed isolates was, however, too low to trace any patterns in diversity and their possible relationships. A further, thorough investigation should involve additional whole-genome sequences, given the possible misidentification of potential recombinant sequences (one of the identified parents might be of recombinant origin; see Supplementary Material).

### 3.5. Transmission Electron Microscopy

**Figure 6:**
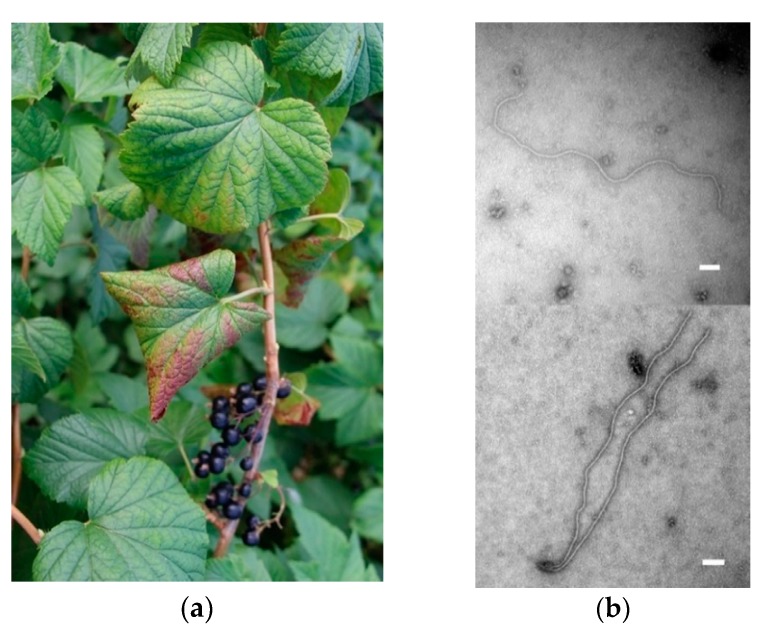
(**a**) Leafroll symptoms on the black currant plant, 28074: downward curling of leaf margins and interveinal red coloration (Switzerland, July 2017); (**b**) individual particles obtained after viral particle enrichment of the black currant 28074 leaf material. The scale bar represents 100 nm.

After particle purification of BC28074-positive leaf material showing leafroll symptoms (Figure 6a), long thread-like particles were visualized (Figure 6b), typical for members of the family *Closteroviridae* [15], with the most frequent length being 1500 nm and the most frequent width being ca. 11 nm (*n =* 125).

## 4. Conclusions

Several diverse closterovirid isolates were identified in the currant in Europe and North America. Sequence analyses of the whole genome, as well as phylogenetic inference, confirmed that they all belonged to a novel species of the genus *Closterovirus*, family *Closteroviridae*, tentatively named blackcurrant leafroll associated virus 1 (BcLRaV-1). The presence of the virus was further confirmed using electron microscopy and via sequencing of RT-PCR amplicons. Sequence comparison of all genes revealed high molecular variability across isolates (Figure 3), with p13, a protein of unknown function, being the least conserved. The phylogenetic analyses of selected proteins revealed topological differences between the trees based on the 1b and CP/HSP70h (Figure 5), potentially presenting the evidence of potential intraspecies recombination events. Indeed, several possible recombination points were located between the black and red currant isolates (Table 3). This may indicate complex transmission routes that enabled the coinfection of a single host by the hypothetical parental genomes in the past. Nevertheless, recombination analysis involving a wider dataset is required to understand the evolutionary process giving rise to the virus genome.

The nearly identical US and SLO isolates (Figure 3) may reflect a long-distance movement of virus-infected *Ribes* plants. Further investigation should evaluate whether or not the divergent isolates have different pathogenicity capacities.

## Figures and Tables

**Table 1:** Origin of currant isolates and the description of high-throughput sequencing (HTS).

BcLRaV-1 Isolate/GenBank Accession Number	Plant	Origin	Symptoms	Sequencing	
				Input	HTS Output
G55/MH460557	Red currant, Gabreta 55	Czech Republic	Asymptomatic	Total RNA: mRNA enriched	20 millions, 100 bp reads
GR/MH460558	Red currant, Gondouin Rouge	Czech Republic	Asymptomatic		
SLO/MH480582	Black currant, unknown cultivar	Slovenia	Asymptomatic	Total RNA: Ribo-depleted	10 millions, 2 × 150 nt reads
BC28074/MH541840	Black currant, 28074	Switzerland	Leafroll [6]	Viral-associated nucleic acid	50 millions, 2 × 75 nt reads
US/MH500053	Black currant, NCGR PI 556169	USA	Yellow line patterns [13]	Enriched double-stranded RNA	76,214 reads

**Table 2:** Genomic characteristics of the BcLRaV-1 isolates.

Isolate	Genome Length (nt)	Genome Elements										
		5′-UTR (nt)	3′-UTR (nt)	Length of ORFs for Predicted Proteins (nt)/ Molecular Mass of Encoded Proteins (kDa)								
				1a/1b	p6	HSP70h	HSP90	CP	CPm	p17	p13	p26
G55	17,313	97	275	9939/371.9	147/5.7	1797/65.6	1593/61	636/23.6	606/22.3	456/16.8	348/13.1	690/25.6
GR	17,161	99	309	9906/370.5	147/5.7	1797/65.5	1590/60.9	636/23.6	606/22.2	441/16.3	348/13.2	690/25.8
SLO	16,894	1 *	260 *	9942/372.7	150/5.6	1797/65.7	1593/60.6	636/23.4	606/22.5	444/16.5	372/14.3	687/25.5
BC28074	17,141	102	290	9942/370.3	150/5.7	1797/65.9	1593/60.4	636/23.5	714/26.7	444/16.4	399/15	687/25.6
US	16,996	99	264	9942/372.7	150/5.6	1797/65.7	1593/60.7	636/23.4	606/22.5	444/16.6	372/14.2	687/25.5

* incomplete sequence, UTR—untranslated region.

**Table 3:** Recombination analysis of BcLRaV-1 isolates.

Predicted Recombinant Isolate	Position of the Recombinant Part (Predicted Breakpoints)	Putative Parental Isolates ^1^		Detection Method								
		Major Parent	Minor Parent	RDP	GENECONV	Bootscan	Maxchi	Chimaera	SiSscan	PhylPro	LARD	3Seq
G55	45–9957	Unknown (US)	GR	1.1 × 10^−52^	NS ^2^	5.1 × 10^−40^	2.2 × 10^−15^	4.6 × 10^-33^	2.3 × 10^-84^	NS ^2^	NS ^2^	1.6 × 10^−5^
US	7701–10,345	BC28074	GR	2.7 × 10^−5^	NS ^2^	2.3 × 10^−2^	1.5 × 10^−4^	7.5 × 10^−6^	5.8 × 10^−4^	NS ^2^	4.3 × 10^-51^	2.2 × 10^−5^
US	4514–4729	GR	G55	1.0 × 10^−3^	2.5 × 10^−2^	7.2 × 10^−3^	NS ^2^	4.3 × 10^−2^	8.5 × 10^−9^	NS ^2^	NS ^2^	4.1 × 10^−2^

^1^ Major and minor parents—predicted contribution of the larger and smaller sequence fragments, respectively, ^2^ NS—no support detected.

## Supplementary Materials

The following are available online at http://www.mdpi.com/1999-4915/10/7/369/s1, Table S1: Recombination analysis of BcLRaV-1 isolates.xslx.
